# Impact of sex hormones on postoperative outcomes in plastic surgery: a narrative review

**DOI:** 10.3389/fsurg.2025.1587708

**Published:** 2025-08-21

**Authors:** Xiong Lv, Chun Xiang, Yan Zheng, Xuling Lv, Wanxuan Zhou, Jiajun Zhu

**Affiliations:** Plastic Surgery Department, The Quzhou Affiliated Hospital of Wenzhou Medical University, Quzhou People’s Hospital, Quzhou, China

**Keywords:** estrogen, testosterone, plastic surgery, skin healing, transgender patients

## Abstract

**Background:**

Since 1929, when scientists first identified estrogen in urine and coined the term “sex hormones,” these vital steroid hormones have been recognized for their critical role in tissue repair and wound healing. This is particularly evident in the postoperative recovery of plastic surgery patients. While the effects of sex hormones differ between males and females, their mechanisms in wound healing, angiogenesis, and collagen regulation have drawn significant attention.

**Methods:**

This narrative review synthesizes key literature from four databases including PubMed (up to 2024) focusing on the effects of sex hormones on healing after plastic surgery.Key terms such as “sex hormones,” “tissue repair,” and “postoperative recovery” were used. The analysis highlights the differential roles of estrogen and testosterone in skin healing, angiogenesis, and inflammatory responses. Additionally, it explores the effects of sex, age, and hormone replacement therapy (HRT) in transgender patients on surgical outcomes.The main target audience of this article is professional surgeons and endocrinologists, medical students and scientific researchers.

**Results:**

Existing evidence suggests that estrogen enhances wound healing by upregulating vascular endothelial growth factor (VEGF), stimulating angiogenesis, and playing a pivotal role in collagen regulation. Testosterone may influence fibroblast proliferation and angiogenesis, although its effects vary by sex and age. Postmenopausal women exhibit diminished healing capacity due to decreased estrogen levels, whereas transgender patients undergoing HRT show improved postoperative recovery.

**Conclusion:**

Sex hormones significantly influence postoperative recovery in plastic surgery. The distinct mechanisms of estrogen and testosterone in wound healing provide valuable insights for personalized medical approaches, optimizing surgical outcomes across diverse patient populations.

## Introduction

Sex hormones, as steroid hormones, have long been studied as critical chemical messengers influencing human physiological functions in the modern era. In addition to defining human sex characteristics, sex hormones play a crucial role in systemic metabolic regulation. Estrogens promote protein synthesis and cell proliferation in target tissues through estrogen receptors (such as ERα/β) and membrane-associated signaling pathways (1) [Level D]. In the skin, estrogen stimulates fibroblasts to synthesize collagen by upregulating the expression of transforming growth factor-β (TGF-β) (2). Similarly, testosterone activates protein synthesis pathways by binding to androgen receptors (AR), enhancing the efficiency of protein synthesis in muscle and skin cells while simultaneously inhibiting protein breakdown (3, 4). These anabolic effects provide a physiological basis for the role of sex hormones in postoperative repair.Sara Merlo et al. described how selectively activating Estrogen Receptor beta(ER β) might positively influence wound healing (5), a factor of paramount importance in plastic surgery.

The human pursuit of beauty has driven the evolution of plastic surgery. As early as 600 BCE in India, texts documented the use of forehead flaps for nasal reconstruction (6). Today, plastic surgery remains a classic treatment for repairing damage to external organs. Concurrently, the rapid development of media and social networks has increased public acceptance of plastic surgery. For instance, in South Korea, there are 13.5 cosmetic surgeries per 1,000 people; in the United States, the number of cosmetic procedures grew to 15.7 million by 2016, marking a substantial increase since the mid-20th century (7, 8).

This review aims to explore the impact of sex hormones on postoperative outcomes in plastic surgery, the mechanisms underlying their effects, and the differences in their roles across various populations and genders. Additionally, it discusses the direction for developing personalized medical approaches to optimize patient care.

## Search strategy

We searched four databases (PubMed, Embase, Cochrane Library, and Web of Science) and the Yandex search engine until June 2024. The language was restricted to English and Russian, and the search strategy included the following keywords: “human sex hormones,” “androgens,” “estrogens,” “plastic surgery,” “Gender-affirming surgery,” “sex,” “thrombosis,” “surgical history,” and “nursing.” There were no restrictions on study design. Initially, we screened the titles and abstracts of the articles for relevance to hormonal mechanisms, postoperative healing, and clinical significance. Key articles were selected, and we subsequently evaluated the full-text versions of the remaining articles. Inclusion was determined by the authors and aimed to highlight representative studies of the main findings and controversies in the field. This narrative review does not infer causation nor claim systematic evidence synthesis. The review did not involve the collection of primary patient data; therefore, ethical approval was not required.

## Evidence grading approach

As a narrative review, this article synthesizes evidence without formal meta-analysis. To enhance transparency, we categorized findings by evidence strength: Level A: Large human studies(Sample size ≥ 200); Level B: Clinical human studies with limitations(Sample size < 200); Level C: Preclinical/animal data; Level D: Mechanistic hypotheses. Annotated all key conclusions with evidence levels in text, the level of evidence is noted in the table. Clinical recommendations prioritize Level A-B evidence.

## Impact and mechanisms of sex hormones on plastic surgery outcomes

### Human sex hormones

**Figure 1:**
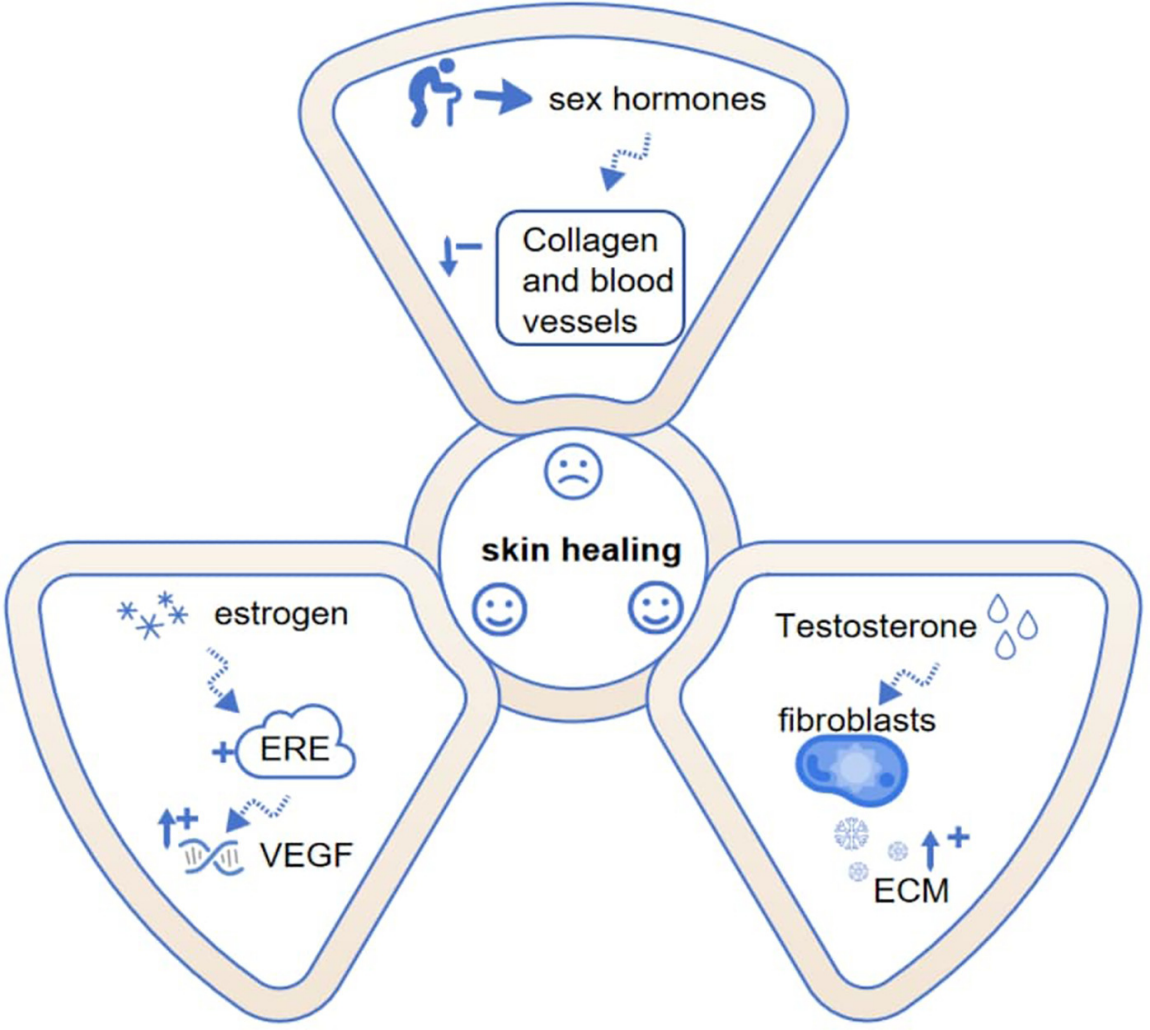
Mechanistic pathways of sex hormones in skin healing. Dashed lines indicate effects, while arrows with “+” and “−” symbols represent enhancement and weakening. Estrogen and testosterone promote healing through distinct mechanisms, though capacity declines with age. ECM, extracellular matrix; VEGF, vascular endothelial growth factor; ERE, estrogen response element.

Sex glands, including the placenta and the zona reticularis of the adrenal cortex, synthesize steroid hormones such as estrogen and testosterone using cholesterol as a precursor. These hormones not only promote sexual organ maturation and maintain secondary sexual characteristics but also play a critical role in tissue repair, as summarized in Figure 1. Testosterone can affect fibroblasts and activate extracellular matrix; Estrogen primarily functions in an endocrine manner, reaching target tissues such as the uterus, vagina, skin, and pelvis via circulation. However, it's also worth noting that in some local microenvironments, like within the ovary, estrogen produced by granulosa cells can act in a paracrine manner to regulate the function of adjacent cells. In these tissues, it binds to specific receptors, playing a significant physiological role in the processes of repair and healing (Figure 1).

### Tissue healing after plastic surgery

Compared to conventional surgical procedures, plastic surgery plays a pivotal role in improving external appearance, particularly in facial surgeries. Since 2022, the proportion of procedures such as blepharoplasty, facelifts, and rhinoplasty has steadily increased in plastic surgery (9). Facial skin is richly vascularized, with the facial artery (a branch of the external carotid artery) supplying blood to regions like the lips, nose, and eyelids. During surgery, damage to local vascular structures reduces perfusion, creating a hypoxic microenvironment. In response to hypoxia, vascular endothelial growth factor (VEGF) is released, binding VEGFR2 receptors to stimulate endothelial proliferation and vascular permeability (10, 11). This process is initiated by hypoxia-inducible factor 1-alpha (HIF-1α) which accumulates when prolyl hydroxylase (PHD)-mediated degradation is inhibited (12). HIF-1α then translocates to the nucleus, dimerizes with HIF-1β, and activates VEGF expression by binding hypoxia response elements (HREs) in its promoter (13), thereby facilitating angiogenesis. Moreover, VEGF enhances HIF-1α activity through positive feedback, synergistically promoting angiogenesis and tissue healing (14).

### Estrogen-related postoperative skin healing

Estrogen rapidly upregulates vascular endothelial growth factor (VEGF) expression, evidenced by a significant increase in VEGF mRNA within one hour of subcutaneous estrogen administration (40 mg/kg) (15). This effect extends transplacentally, as demonstrated by elevated fetal skeletal muscle VEGF and enhanced microvascular development in baboons following maternal estrogen injection (16) [Level C]. The systemic vascular regulatory role of estrogen—mediated through classical nuclear signaling where estrogen dimers activate the VEGF gene's estrogen response element (ERE) (17–19) [Level D]—confirms its mechanistic consistency across multiple target tissues including skin, uterus, heart, and bone.

Collagen is an essential structural matrix of the skin, responsible for regulating its mechanical properties and elasticity (20). Collagen secretion and estrogen levels peak during early adulthood (ages 20–30), demonstrating a strong correlation (21). Postmenopausal women experience a yearly reduction of approximately 1.13% in skin thickness and a 2% annual decline in collagen content (22). Tissue damage from plastic surgery activates matrix metalloproteinase-9 (MMP9), which rises significantly within 12 h post-surgery. MMP9 degrades the extracellular matrix (ECM), facilitating keratinocyte migration and accelerating wound healing (23). However, excessive MMP9 activity may hinder the formation of new tissue (24).

Estrogen protects tissues by inhibiting MMP9 activity, maintaining it at an appropriate level (25). Additionally, estrogen stimulates the production of type I and III collagen and fibrinogen, preserving skin thickness (26). Certain phytochemicals, such as resveratrol, exhibit estrogen-like effects by upregulating Tissue Inhibitor of Metalloproteinases-1(TIMP1), inhibiting MMPs activity, and increasing collagen and elastin synthesis, thus preventing collagen degradation (27–29). However, excessive collagen deposition may lead to scar formation, which is associated with hyperactive fibroblasts and increased vascular density (30, 31).

### Androgen-related postoperative skin healing

Testosterone significantly influences tissue healing. A recent study demonstrates that supraphysiological doses accelerate early wound healing in castrated male rats (32) [Level C]. Fibroblasts—key cells secreting extracellular matrix (ECM) to support skin integrity against mechanical stress (33, 34)—interact with ECM via integrins and matrix receptors to regulate cellular responses (35). Testosterone further modulates fibroblast proliferation and ECM remodeling through synergistic actions with insulin-like growth factors (36) [Level D]. This dynamic balance underpins testosterone's role in granulation tissue formation, where subsequent angiogenesis is critical (37). Evidence indicates castrated mice exhibit impaired angiogenesis (38) [Level C], while testosterone metabolites (e.g., 17β-estradiol) maintain skin perfusion (39) [Level C], confirming its multifaceted repair function.

Interestingly, different studies have reported conflicting conclusions regarding testosterone's role in wound healing. Some suggest that testosterone may negatively affect tissue repair (40). For instance, older males often heal more slowly than their female counterparts, possibly due to testosterone exacerbating inflammatory responses during healing (41, 42). These findings suggest that testosterone's impact on wound healing may depend on factors such as age, hormone levels, and its regulatory capacity on inflammation.

### Plastic surgery reshapes sex hormones

While sex hormones critically influence surgical outcomes, emerging evidence suggests that plastic surgery itself may actively reshape endocrine homeostasis, creating a feedback loop that extends beyond the operating room. Forces work against each other—not only in physics but also in the interplay between sex hormones and plastic surgery. Bariatric surgery exemplifies this interaction. A previous study indicated that women who underwent bariatric surgery (vertical banded gastroplasty) and lost 40% of their initial weight experienced significant reductions in estradiol and total testosterone (43). Another prospective cohort study on bariatric surgery revealed that not only did women's sex hormone levels change post-surgery, but there was also a significant improvement in their sexual arousal, lubrication, desire, and satisfaction. Given that collagen synthesis requires local estrogen intervention, surgery also impacts body shape (44). This outcome may be linked to fat loss and alterations in intestinal hormones. The reduction of adipose tissue diminishes aromatase activity, thereby decreasing the conversion of androgens to estrogens, while surgery modifies the secretion of intestinal hormones (such as glucagon-like peptide-1), which may indirectly regulate sex hormone levels while also affecting appetite (45). Notably, some surgical procedures can reverse hormonal imbalances. Patients with gynecomastia may experience sex hormone imbalances and sexual dysfunction. A 2024 study demonstrated that 32 patients who underwent gynecomastia surgery exhibited significantly decreased estrogen levels post-surgery (*p* < 0.001), attributed to the removal of excess breast tissue. Simultaneously, the surgery alleviated the embarrassment associated with excessive breast development, thereby significantly enhancing sexual function assessments (46)[Level B]. These research findings underscore the importance of postoperative monitoring of sex hormones and necessitate that doctors consider the impact on patients' sex hormone levels when formulating surgical plans.

### Men and women differ in how their skin heals after surgery

The impact of different genders on skin healing is complex, contributing to uncertainties in plastic surgery outcomes. Generally, males of all age groups have thicker skin on average compared to females (47) [Level B]. An earlier study attributed this to higher testosterone levels in males, which promote the production of collagen and elastic fibers in the skin (48). However, another study found that after 24 weeks of topical estrogen application on the face, the natural skin thickness (dermis plus epidermis) of women aged 52–70 increased (49), suggesting that testosterone's positive effect on skin thickness may outweigh that of estrogen.

Regarding skin healing, the mechanisms of testosterone appear to be more intricate than those of estrogen. For example, under the influence of testosterone, males often exhibit faster healing of oral mucosal wounds but slower healing of dermal wounds (50–52)[Level A]. Immune responses play a significant role in this process. In male mice, castration has been shown to markedly accelerate local skin wound healing (53). Similar results were observed in hairless mouse models, likely due to testosterone's suppression of macrophage production of tumor necrosis factor-α(TNF-α), which reduces systemic TNF-α levels and suppresses immune function (54). This immunosuppressive effect directly impacts the immune cell population in the wound, particularly macrophages, prolonging the transition to the proliferative phase and resulting in slower wound healing outcomes (Table 1).

**Table 1:** Evidence summary: differential effects of androgens and estrogens.

Sex hormones	PMID	Intervention content	Key findings	Level of evidence
Androgens				
	9034651	Exploring the Physiological Differences in Skin Between Men and Women. (48)	Due to higher levels of testosterone in men, the production of elastin and collagen fibers in the skin is also increased.	
	8409184	Measure plasma insulin-like growth factor-I, total testosterone, and free testosterone in healthy young males, healthy elderly males, and long-term institutionalized elderly males. (56)	As part of the aging process, men's testosterone levels progressively decline after the age of 40.	Level B
	35355085	Forty rats were divided into groups, subjected to either testicular removal or weekly administration of supraphysiological doses (250 mg/kg) of exogenous testosterone, and full-thickness skin excision wounds were created to evaluate wound healing. (32)	Supraphysiological doses of testosterone supplementation promote angiogenesis to enhance skin wound healing.	Level C
	27009546	Three skin wounds were created on the backs of diabetic model rats, and tissue was harvested from different wounds every 7 days. The proportion of collagen fibers in the tissues of rats treated with testosterone was studied. (37)	Rats treated with testosterone exhibited better healing patterns, with wounds completely closed.	Level C
	24157428	Male and female mice were gonadectomized and lesions were collected to assess the density of myofibroblasts, collagen deposition, and the number of blood vessels. (38)	The wound healing of ovariectomized female mice was affected, while the wound healing of castrated male mice remained unaffected.	Level C
	28360090	The effect of testosterone therapy on skin necrosis in castrated male mice. (39)	Testosterone ameliorates skin necrosis in male mice and exerts its effects through its estrogen and androgen derivatives.	Level C
	17276202	The Regulatory Role of Androgens in Skin Wound Healing. (40)	When the balance between estrogen and androgen changes, the effects of testosterone can be harmful.	
	14652627	Summary and exploration of androgen-regulated tissue repair processes. (42)	Androgens promote skin healing by regulating the processes of inflammation and tissue repair.	
	35328552	Exploring the Role of Sex Hormones in the Function of Keratinocytes in Inflammatory Skin Diseases. (52)	The research findings summarize cellular mechanisms and molecular effectors, which can guide the formulation of therapeutic interventions.	
	10770208	Exploring the pathways by which testosterone affects fibroblast proliferation (36)	Testosterone influences fibroblast proliferation and extracellular matrix (ECM) remodeling through its synergistic action with insulin-like growth factors.	Level D
Estrogen				
	7799828	A randomized, double-blind study involving 54 women with moderate to severe facial skin aging was conducted to examine the skin condition after the application of a cream containing estrogen or a placebo cream. (49)	Topically applied estrogen can increase skin thickness.	Level B
	24194966	The Physiology of Estrogen's Impact on Skin Aging. (60)	Postmenopausal skin ages faster and becomes thinner.	
	37810217	A cross-sectional study on estrogen use and VTE occurrence among the female population of British-South Asian descent. (88)	Patients with obesity, hypertension, dyslipidemia, or chronic kidney disease are more susceptible to venous thromboembolism when using estrogen.	Level A
	22776819	Hematopoietic chimeric mice with selective deficiency of estrogen receptor (ER) α or β.Administer high physiological levels of estradiol. (93)	Chronic estradiol treatment reduces the risk of thromboembolism in mice while decreasing platelet reactivity.	Level C
	19008331	Investigate the expression of estrogen receptors in the human keratinocyte cell line NCTC 2544, study cell proliferation using estradiol, and validate the findings through transwell migration assays. (5)	Within hours of estrogen treatment, cell migration accelerates, stimulating cell proliferation and thereby exerting a positive impact on *in vitro* wound healing.	Level D
	11035983	Estrogen's effect on the expression of vascular endothelial growth factor (VEGF) mRNA in tissues of rodents and humans. (15)	Estrogen can rapidly regulate the expression of VEGF, with a significant increase in VEGF mRNA expression observed within 1 h after drug administration.	
	38738915	The impact of estrogen deprivation in maternal baboons on VEGF expression in skeletal muscle, capillary development, and long-term vascular and metabolic functions in baboon fetuses. (16)	The maternal baboon did not receive estradiol treatment, resulting in a 45% suppression of VEGF protein expression in skeletal muscle and a 47% reduction in the number of capillaries in the fetal baboon's muscle fiber area.	Level C
	33339644	The identity and function of membrane receptors in the rapid actions of estrogen. (17)	The signal emitted by mESR1 is a critical contributing factor to the normal estrogen response in the body and influences human fertility.	Level D
	10995484	Through the new model system, the human VEGF promoter-luciferase reporter construct was transiently transfected into primary human endometrial cells and Ishikawa cells to investigate whether estrogen has a direct transcriptional effect on VEGF gene expression. (19)	The estrogen-regulated VEGF gene transcription requires the variant ERE located 1.5 kb upstream of the transcription initiation site.	Level D
	27521253	The Mechanism of Phytoestrogens' Impact on Skin Aging. (21)	Phytoestrogens reduce skin aging by diminishing oxidative stress cascade events through various molecular actions.	
	34044023	Estrogen affects skin aging through biomechanics. (22)	The use of estradiol can prevent the decline in skin structure and mechanical properties associated with aging.	
	19668239	Peripheral blood mononuclear cells (PBMCs) were collected from the spleens of three multiple sclerosis patients treated with estriol and EAE mice, and the levels of matrix metalloproteinases were analyzed. (25)	Estriol enhances MS lesions and EAE inflammatory lesions by reducing MMP-9 in immune cells through ERalpha.	Level C
	30997378	The role of estrogen in the skin and a review of alternative approaches to systemic estrogen therapy. (26)	Low estrogen levels can lead to skin aging in women, and topical estrogen application can improve this condition.	
	18254804	The Phytohormone Effect of Resveratrol. (27)	Resveratrol can significantly enhance antioxidant activity and prevent photoaging.	
	31747092	Exploring the effects of phytoestrogens on human skin and the mechanisms of alleviating aging. (28)	Phytoestrogens can exhibit either agonist or antagonist estrogenic properties in tissues and exert anti-aging effects on the skin.	
	18477406	Using the Affymetrix microarray-based approach, the relationship between estrogen, aging, and delayed wound healing was determined. (41)	83% of the downregulated probe sets and 80% of the upregulated probe sets are estrogen-regulated, indicating that estrogen has a significant impact on aging.	Level C
	11822783	Rats receiving estrogen injections were subjected to burn injuries, and then blood samples were collected for analysis. (53)	The increase in estrogen levels inhibits the elevation of serum TNF-α induced by burns, which may contribute to the amelioration of inflammatory responses caused by burns.	Level C
	37738797	Summarize the impact of estrogen on the NLRP3 inflammasome. (63)	Low estrogen levels lead to excessive activation of the NLRP3 inflammasome, inhibiting skin healing.	
	31774863	Full-thickness wounds were created in female C57BL/6J mice, and one group of the mice was treated with topical estrogen. (64)	Local estrogen therapy can promote wound healing by reducing wound area and inflammatory response.	Level C
	20590611	Investigate the effect of genistein aglycone on skin healing in rats after ovariectomy. (65)	Genistein aglycone promotes wound healing and enhances tensile strength.	Level C
	7968118	By comparing 155 premenopausal women with venous thromboembolism and no other underlying conditions to 169 population controls, the risk of VTE among oral contraceptive users was investigated. (67)	Young women with factor V Leiden mutation who use oral contraceptives have higher risk of venous thrombosis.	Level A

## Effects of menopausal estrogen changes on postoperative wound healing

### Aging and skin

One of the core objectives of medicine is to enhance human quality of life, with age playing a critical role in this process. Research indicates that wound healing capacity declines with advancing age, which is associated with increased mortality and a higher risk of postoperative complications. For example, studies have shown that the incidence of postoperative complications following free flap transplantation surgery is significantly higher in elderly patients (55), highlighting the profound impact of age on recovery outcomes.

### Skin healing during menopause related to sex hormones and nutrition

In males, testosterone levels typically begin to decline gradually after the age of 40, closely linked to the aging process (56). In females, menopause occurs between the ages of 40 and 60, characterized by the depletion of ovarian follicular reserves and reduced ovarian responsiveness to gonadotropins, leading to a significant decrease in sex hormones such as estrogen (57, 58)[Level A]. Postmenopausal women often experience symptoms such as insomnia and mood swings, and the decline in estrogen levels has a profound impact on skin characteristics and skin healing ability (Table 1).

As the most estrogen-responsive non-reproductive organ in the body, the skin becomes thinner and more prone to dryness after menopause (59, 60). These changes are closely associated with reduced sebaceous gland activity and collagen loss. Additionally, low estrogen levels promote the production of interleukin-6 (IL-6) and suppress macrophage anti-inflammatory activity in response to lipopolysaccharide and interferon-*γ* stimulation, increasing the risk of inflammation. In some cases, low estrogen levels may also lead to overactivation of the NOD-like receptor thermal protein domain associated protein 3(NLRP3) inflammasome, exacerbating inflammatory responses and inhibiting skin healing (61–63). Thus, postmenopausal women face unique challenges in postoperative skin healing.

Several intervention strategies have been explored to address these issues. In addition to traditional systemic hormone replacement therapy (HRT), topical estrogen application has demonstrated promising efficacy. An animal study demonstrated that, for full-thickness wounds approximately 4 mm in diameter in 80-week-old female mice, the elderly group treated with topical estrogen exhibited significant advantages over the elderly group treated with vehicle wound therapy. This treatment notably reduced the wound area and inflammation, thereby accelerating the wound healing process (64) [Level C]. Moreover, phytoestrogens, such as genistein, have been identified for their anti-inflammatory properties and are thought to effectively improve wound healing in women with declining estrogen levels. Even at low doses, genistein significantly increases the levels of vascular endothelial growth factor and transforming growth factor-β1 in wounds, while enhancing the skin's resistance to rupture (65) [Level C].

Beyond hormonal interventions, adequate nutritional supplementation is equally essential. Key nutrients such as vitamin C, vitamin E, and omega-3 fatty acids play a vital role in reducing postoperative bruising, swelling, and other adverse reactions (66). However, whether these nutrients synergize with hormonal therapies to further enhance postoperative recovery remains an area requiring further investigation.

## Genetic and environmental factors and patient response to hormones

When patients undergo hormone therapy, genetic factors can influence its effectiveness. Research by Vandenbroucke et al. indicates that genetic factors may modulate the beneficial and adverse effects of estrogen. For instance, women with coagulation factor V Leiden mutations who take oral contraceptives face a thrombosis risk about 30 times higher than that of non-carriers not using such contraceptives (67) [Level B]. Certain gene mutations, like those in the ESR1 gene, can render individuals completely unresponsive to estrogen. These mutations typically impair receptor function, leading to lost estrogen signaling and cellular insensitivity to both endogenous and exogenous estrogen, thus impacting estrogen's effects (68).

Beyond genetics, environmental factors also shape patients' hormone responses in various ways. The natural environment is rife with endocrine—disrupting chemicals (EDCs) that can mimic or interfere with natural hormones. For example, some plastics (e.g., bisphenol A) and pesticides may disrupt estrogen function, potentially affecting wound healing and even causing cancer (69). Human activities impact the environment, which in turn affects us. Studies show that artificial light and electromagnetic waves can influence ovarian steroid hormone secretion, possibly via the hypothalamus—pituitar*y* axis (70). Although research in this area is still limited, based on existing findings, we have reason to believe that external environmental factors can impact hormone therapy effectiveness, warranting more in-depth investigation.

## Transgender patients: the effects of hormone replacement therapy HRT on surgical outcomes for gender-confirmation surgeries

### Transgender individuals

According to the World Professional Association for Transgender Health (WPATH), transgender patients encompass all individuals whose gender identity is inconsistent with their sex assigned at birth. MTF (Male-to-Female) refers to transgender individuals who identify as female (assigned male at birth); FTM (Female-to-Male) refers to transgender individuals who identify as male (assigned female at birth). In recent years, the number of transgender individuals, particularly among adolescents and young adults, has significantly increased (71). This population often experiences social, psychological, and physiological stressors, leading many to seek gender-affirming medical interventions, including hormone HRT and gender-affirming surgeries.

### Hormone therapy for transgender individuals

HRT is a cornerstone of transgender healthcare, involving the administration of exogenous hormones (such as estrogen or testosterone) to align physical characteristics with an individual's gender identity. For male-to-female (MTF) patients, estrogen and anti-androgens are the primary treatments. Estrogen binds to estrogen receptors in the body, promoting breast development and other feminizing characteristics, while anti-androgens inhibit testosterone activity, reducing masculine traits (72). For female-to-male (FTM) patients, testosterone therapy binds to androgen receptors, activating the expression of genes associated with masculinization and promoting the development of male secondary sexual characteristics. Additionally, estrogen inhibitors are used to suppress the effects of endogenous estrogen, further aiding in masculinization (73).

### Impact of sex hormones in gender-affirming surgery patients

Gender-affirming surgery, defined as surgery aimed at reconciling physiology with gender identity (e.g., mastectomy, phalloplasty), presents unique physiological demands, with preoperative hormone replacement therapy (HRT) playing a key role in optimizing surgical outcomes by regulating tissue quality and enhancing postoperative healing. Preoperative Hormonal Preparation: HRT is essential for aligning patients' hormonal status with surgical goals. In male-to-female (MTF) patients, estrogen is associated with breast tissue development and increases dermal thickness and elasticity, which are critical for procedures requiring precise skin incisions (e.g., breast augmentation) (74). In female-to-male (FTM) patients, testosterone correlates with muscle hypertrophy and extracellular matrix (ECM) remodeling, thereby improving structural integrity during phalloplasty. Animal models have demonstrated that testosterone supplementation at supraphysiological doses can improve and accelerate granulation tissue maturation, promote early-stage skin wound healing, and enhance granulation tissue maturation (32) [Level C]. Furthermore, clinical studies have reported a lower rate of wound dehiscence in FTM patients receiving testosterone HRT, which is attributed to enhanced fibroblast activity (75).

Postoperative Healing and Recovery: Hormone Replacement Therapy (HRT) sustains benefits during recovery by improving tissue perfusion and reducing inflammation. Estrogen is linked to microvascular perfusion at surgical sites, thereby reducing the risk of necrosis. For instance, male-to-female (MTF) patients undergoing mastectomy while on HRT exhibit accelerated healing due to improved oxygenation and nutrient delivery (76, 77). Testosterone, on the other hand, suppresses inflammatory cytokines (e.g., TNF-α) in female-to-male (FTM) patients, facilitating a smoother transition to the proliferative healing phase (78) (Table 2).

**Table 2:** Characteristics of transgender patients (MTF/FTM).

Transgender Patients	PMID	Intervention content	key findings	Level of evidence
Male to Female				
	31656099	The Risks and Mechanisms of Hormone Affirmation Therapy for Transgender Individuals. (72)	For patients with MTF, use both estrogen and anti-androgens to promote feminizing features.	
	33753543	The estrogen and testosterone usage of 611 transgender individuals was followed up. (92)	Transgender individuals using physiological doses of gender-affirming hormone therapy are not at risk of thrombosis in the short term.	Level A
	39382481	The wound healing process following gender-affirming surgery for transgender individuals. (76)	Patients who underwent HRT healed faster after mastectomy than those who did not receive HRT.	
	38138197	The impact of sex hormones on the health maintenance of transgender individuals was explored. (77)	Estrogen can improve blood circulation and nutrient delivery to the wound site, thereby accelerating the healing process.	
	29756046	This study analyzed data from transgender women treated in the clinic since 2007, gradually increasing the dose of estrogen from low to high over a period of one year and measuring and evaluating the success rate of returning serum testosterone and 17-β-estradiol levels to within the normal range. (84)	Oral estradiol can attain the necessary serum levels of the drug; however, individual dosage variations exist, and oral estradiol alone frequently fails to provide sufficient testosterone suppression.	Level A
	30602475	Thrombotic risk in transgender women receiving estrogen therapy. (89)	The overall estimate of incidence in this population was 2.3 cases/year/1000 people (95% CI, 0.8–6.9).	
Female to Male				
	37476490	Pharmacodynamics, Comparative Dosage, Adverse Reactions, and Pharmacogenetic Effects of Gender Transition Medications. (73)	For patients with FTM, while using androgens, estrogen inhibitors can inhibit the effects of endogenous estrogen and further virilization.	
	37822706	The incidence of thrombosis and changes in plasma components among the transgender population were discussed, and three cases were presented for further investigation. (94)	As part of a comprehensive approach to gender-affirming care, the treatment for different patients should be individualized. Physicians with knowledge of dynamic coagulation testing need to be involved in the clinical management.	

Risk-benefit considerations: Although HRT can enhance recovery efficacy, its associated risks necessitate personalized management. Patients undergoing transgender surgery or other specialized surgical procedures are often in a hypercoagulable state due to factors such as postoperative bed rest, slow blood flow, and surgical trauma. Prolonged use of sex hormones may further elevate the risk of thromboembolism (e.g., pulmonary embolism). Therefore, preoperative evaluation, medication adjustment, and postoperative anticoagulation therapy are essential for these patients.

### Long-term outcomes of hormone therapy

In addition to its physical benefits, HRT significantly improves psychological well-being. Many patients report enhanced emotional health and social functioning after initiating HRT, with marked improvements observed within six months of starting treatment (79) [Level B]. HRT also effectively alleviates gender dysphoria, helping patients achieve a greater sense of self-alignment. Studies have shown that patients receiving HRT report significantly higher body image satisfaction and lower levels of gender-related anxiety compared to those who do not undergo HRT (80). Among transgender and nonbinary adolescents, HRT suggests a protective psychological effect, significantly reducing suicide risk (81).

Overall, HRT not only facilitates the physiological preparation and recovery from gender-affirming surgeries but also enhances patients' psychological health and overall quality of life. This comprehensive treatment approach improves surgical success rates and significantly enriches the long-term life experiences of transgender individuals. However, further research is warranted to evaluate the adverse effects and long-term outcomes of HRT.

It should be pointed out that varying hormone treatment plans and treatment durations can impact surgical results. Appropriately arranged hormone therapy can keep sex hormone levels within the normal range following gender confirmation surgery while curbing the body's natural production of sex hormones (82). Generally, transgender women need to use estrogen for an extended period to maintain typical female characteristics. The recommended oral dose of 17-β estradiol for transgender women who have not had an orchiectomy is around 2–6 mg (83, 84), whereas patients who have had the surgery can take about 1–5 mg, which shows that individual differences lead to differences in hormone therapy. In addition to dosage, the length of hormone treatment can influence self-identity after gender transition, such as the transgender voice. Even though some transgender men rely on surgery to address physiological changes, the anatomical structure of their larynx has not changed, and there is no significant change in the resonance and pitch of their voice (85). Prolonging the duration of hormone treatment helps improve this issue. A study on the self-perception of voice in transgender individuals showed that after 12 months of testosterone treatment, the pitch of transgender men decreased and produced a significant masculinization effect (86). Overall, these findings indicate that when conducting hormone therapy, changes in treatment plans, treatment duration, or individual metabolic differences must be considered, all of which can significantly impact surgical outcomes.

### Adverse effects of hormone therapy

Hormone therapy, while effective in many cases, is not without its drawbacks. A significant concern is the potential for adverse effects, which can include fatigue, loss of libido, and digestive system disorders. One of the most severe side effects is thromboembolism induced by estrogen. In general, patients undergoing abdominal plastic surgery, particularly those with obesity and longer operating times, face an increased risk of deep vein thrombosis (DVT). This risk is associated with hypercoagulability and potential flap failure. Treatment with a specified dose of enoxaparin has been shown to reduce the risk of venous thromboembolism (87). However, the use of estrogen in patients may complicate this scenario. A recent cross—sectional study of women in the British—South Asian cohort found that women with underlying conditions such as obesity, hypertension, and chronic kidney disease had an increased risk of venous thromboembolism when using estrogen (88). This finding is consistent with a previous large-scale case-control study, suggesting that estrogen may be an independent risk factor for venous thromboembolism.

For transgender patients, the risks associated with hormone therapy are particularly noteworthy. Transgender women who receive estrogen treatment are at a higher risk of thromboembolism due to the long-term nature of their hormone therapy. A meta-analysis has shown that transgender women receiving estrogen replacement therapy have a higher risk of venous thromboembolism (89). Combined oral contraceptives (COCs) and systemic hormone replacement therapy (HRT) significantly increase the risk of deep vein thrombosis (DVT) by 2–3 times, primarily due to the effects of estrogen on the synthesis of coagulation factors in the liver (90, 91). Patients who are taking oral contraceptives and require elective surgery, along with prolonged bed rest, may face an elevated risk of thrombosis. Therefore, it is essential to discontinue the use of oral contraceptives three to four weeks prior to surgery to mitigate the risk of pulmonary embolism and mortality. In contrast, existing case reports and retrospective studies have indicated that the use of testosterone in transgender men does not appear to increase the risk of thrombosis (92). Interestingly, an animal experiment once demonstrated that chronic estradiol treatment could protect mice from thromboembolism by reducing platelet reactivity (93) [Level C]. This finding suggests that estrogen may have some unrecognized effects on thromboembolism. We can use *in vitro* coagulation data to help manage thromboembolic risk in transgender individuals (94).

In summary, while hormone therapy has significant benefits for wound healing and skin regeneration, the potential side effects, especially the risk of thromboembolism associated with estrogen use, should be carefully considered. Future research should continue to explore strategies to minimize these side effects and ensure the safe and effective use of hormone therapy.

### Gender-affirming surgery patients affected by lack of information

Despite the growing awareness of gender-affirming surgery, patients continue to experience significant information gaps. This lack of knowledge arises from systemic barriers, including unreliable information sources and insufficient communication with healthcare providers. A recent evaluation of 20 medical websites utilizing a modified EQIP scale yielded an average score of 22.5, which falls short of the standard score of 26. This finding highlights critical deficiencies: only 10% of the websites provided detailed descriptions of the surgical process, associated risks, and potential complications, leaving patients ill-equipped to navigate the challenges of postoperative recovery. Furthermore, only 15% of the websites referenced peer-reviewed studies, which diminishes their credibility. These shortcomings exacerbate the existing barriers faced by transgender patients (95). The Internet should not be relied upon as a primary source of medical information. It is essential for healthcare providers to elucidate the complexities of gender-affirming surgery to patients, while the information available online must be meticulously curated and vetted by experts. Reliable data, articulated in clear language, should be presented on online platforms. Comprehensive information regarding complications should also be included to ensure patients have a complete understanding. Only in this manner can patients access high-quality information that supports their decision-making and promotes appropriate use of Internet resources.

## Research heterogeneity, limitations, and future directions

This narrative review aims to synthesize key ideas rather than systematically assess all available evidence. The selection of studies was based on their relevance to the topic and the authors' judgment, consequently, the literature search was not exhaustive and may have been subject to selection bias, potentially affecting the interpretation of the findings. Firstly, differences existed in the patient demographics across the studies, encompassing variations in age, gender, and baseline health conditions. Such disparities can impact the broader applicability of the results, as different patient populations may exhibit distinct responses to hormone therapy. For instance, younger patients may demonstrate differing hormonal levels and recovery capacities in comparison to older patients, which might interact with the effects of hormonal treatment. Additionally, the surgical techniques utilized were not consistent, with some studies favoring minimally invasive methods while others employed traditional open surgical techniques. The level of tissue trauma and inflammatory reaction associated with varying surgical strategies can affect postoperative recovery and the effectiveness of hormone therapy. Minimally invasive surgery may result in less tissue damage and a quicker recovery, which could modify the impact of hormone therapy on the healing process. Finally, the ways in which hormones were administered varied among studies, including routes like oral, topical, and injectable formulations. The method of hormone delivery can affect their absorption, distribution, and metabolism, ultimately influencing the treatment outcomes. For example, topical administration might provide more localized effects, while oral routes could lead to systemic responses that affect the entire body. This research presents several limitations: most of the cited studies are correlational and do not establish direct causal relationships. The causal effects of hormones on surgical outcomes warrant further investigation. Confounding factors, such as baseline health, surgical techniques, and variations in immune responses, may interact with hormonal treatments and influence the results. It is important to note that the potential cumulative effects of long-term hormone treatment on wound healing, immune response, and tissue integrity warrant further investigation. Future studies should address these limitations by employing well-designed prospective cohort studies and randomized controlled trials to determine the direct effects of hormones. While we graded evidence quality using a narrative approach, future systematic reviews with standardized tools (e.g., GRADE) may provide higher certainty for clinical guidance. Additionally, studies should control for confounding factors and focus on differences in patient responses based on genetic and environmental influences. Genetic variations and environmental factors, including diet, lifestyle, and exposure to toxins, can significantly affect hormone metabolism and response. Finally, research is needed to investigate the impact of different surgical techniques and hormone administration methods on healing to identify the most effective strategies. In summary, while this review provides insights into the role of hormones in postoperative healing, significant limitations persist. Future research should aim to establish causality, understand variations in patient responses, and explore different surgical and hormonal approaches to enhance the quality of evidence and treatment protocols.

## Conclusion

Current evidence suggests sex hormones significantly influence plastic surgery recovery through distinct biological pathways. Topical estrogen formulations (e.g., 17β-estradiol gel) may help counteract collagen depletion and enhance angiogenesis in menopausal and transgender patients. Testosterone's effects appear context-dependent: while potentially beneficial for granulation tissue maturation in phalloplasty, its pro-inflammatory tendencies in elderly males could warrant consideration of targeted anti-androgen strategies. Given documented thrombotic risks with estrogen, preoperative hormonal assessment combined with enoxaparin prophylaxis might be prudent for high-risk surgical cohorts.

Potential future clinical approaches could explore: rapid hormone receptor assays for reconstruction planning, adaptable hormone therapy regimens, and nutrient combinations like genistein/ascorbate to support tissue repair. Research gaps highlight the need for better mapping of cutaneous hormone receptors and integration of endocrine biomarkers into surgical planning.

These findings imply surgeons should consider endocrine profiles during treatment planning. Attention to these biological factors may contribute to improved functional and aesthetic outcomes beyond basic wound healing.
